# Role of Decompressive Craniectomy in the Management of Cerebral Venous Sinus Thrombosis

**DOI:** 10.3389/fneur.2019.00511

**Published:** 2019-05-15

**Authors:** Raghunath Avanali, M. S. Gopalakrishnan, B. Indira Devi, Dhananjaya I. Bhat, Dhaval P. Shukla, Nagesh C. Shanbhag

**Affiliations:** ^1^Department of Neurosurgery, Government T. D. College, Allapuzha, India; ^2^Department of Neurosurgery, Jawaharlal Institute of Postgraduate Medical Education and Research, Pondicherry, India; ^3^Department of Neurosurgery, National Institute of Mental Health and Neurosciences, Bangalore, India; ^4^NIHR Global Health Research Group on Neurotrauma, University of Cambridge, Cambridge, United Kingdom

**Keywords:** anticoagulation, cerebral venous sinus thrombosis, decompressive craniectomy, outcome, risk factors

## Abstract

Cerebral venous sinus thrombosis (CVST) is a relatively uncommon cause of stroke more often affecting women and younger individuals. Blockage of the venous outflow rapidly causes edema and space-occupying venous infarctions and it seems intuitive that decompressive craniectomy (DC) can effectively reduce intracranial pressure just like it works for malignant middle cerebral artery infarcts and traumatic brain injury. But because of the relative rarity of this type of stroke, strong evidence from randomized controlled trials that DC is a life-saving procedure is not available unlike in the latter two conditions. There is a possibility that other forms of interventions like endovascular recanalization, thrombectomy, thrombolysis, and anticoagulation, which cannot be used in established middle cerebral artery infarcts and TBI, can reverse the ongoing pathology of increasing edema in CVST. Such interventions, although presently unproven, could theoretically obviate the need for DC when used in early stages. However, in the absence of such evidence, we recommend that DC be considered early as a life-saving measure whenever there are large hemorrhagic infarcts, expanding edema, radiological, and clinical features of impending herniation. This review gives an overview of the etiology and risk factors of CVST in different patient populations and examines the effectiveness of DC and other forms of interventions.

## Introduction

Cerebral venous sinus thrombosis (CVST) is a stroke caused by blockage of cortical veins and dural venous sinuses which leads to infarction of the draining zone brain parenchyma. It manifests as headache (in 75–95% of cases), seizures, papilledema, altered consciousness, and focal neurological deficits (1–4). CVST is the least common form of acute cerebrovascular disease, accounting for just 0.5% of all types of stroke (5–7). However, this figure rises to 15% of all young strokes in the Asian population (8, 9). The commonest site of origin of thrombosis is believed to be the junction of cerebral veins and larger sinuses (10). Several disorders can cause or predispose patients to CVST such as genetic and acquired prothrombotic disorders, cancer, hematological diseases, vasculitis, systemic inflammatory disorders, pregnancy, puerperium, and infections. In addition, there are a number of local causes such as brain tumors, arteriovenous malformations, basilar skull fracture, CNS infections, and extracranial infections like those arising from the ear, sinus, mouth, face, or neck (11–14). Medical or surgical conditions that increase the likelihood of deep vein thrombosis also increase the risk of intracranial venous thrombosis. In the international prospective study on the cerebral vein and dural sinus thrombosis (ISCVT), 44% of the patients had >1 risk factors. Congenital or genetic thrombophilia was present in 22% of patients. In about 13% of adult CVST patients, despite an extensive search, no underlying risk factors could be found (15). More than 90% of the CVST occurs in people below 60 years of age and it is more commonly seen in women between 20 and 35 years. Young women have a higher risk due to pregnancy, puerperium, and oral contraceptive usage (16–20). The incidence of CVST is estimated to be 1–13 cases per 100,000 per year (5, 16, 21). The incidence in neonates and children is 0.67 cases per 100,000 children, and that of perinatal CVST is 11.6 per 100,000 deliveries in pregnant women (16, 22, 23).

## Decompressive craniectomy in CVST

### Indications

**Figure 1:**
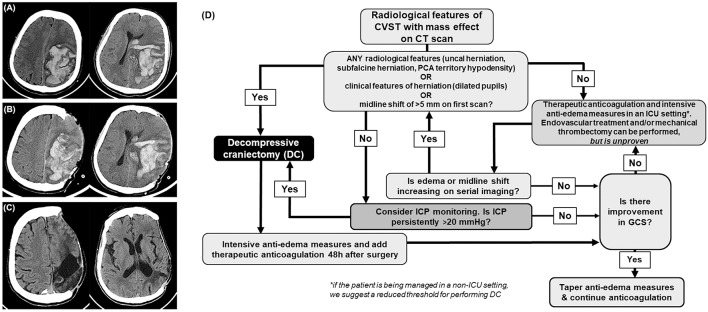
Cerebral venous sinus thrombosis (CVST). **(A)** Computed tomography depicts a confluence of blotchy areas of bleed typically seen in hemorrhagic CVST with mass effect. **(B)** Day 1, and **(C)** 8 months, post decompressive craniectomy. **(D)** Flowchart outlining the management of CVST. CT, computed tomography; ICU, intensive care unit. GCS, Glasgow coma scale; ICP, intracranial pressure.

About 4% of the patients develop supratentorial parenchymal hemorrhagic lesions and cerebral edema severe enough to cause brain herniation and deterioration in neurological functions (8). The term “malignant CVST” is often used to designate this entity (24). Although anticoagulation to promote recanalization by preventing thrombosis progression is considered the mainstay of CVST treatment, it is insufficient to treat the ongoing mass effect of a malignant CVST (24). When aggressive medical management fails to control the raised intracranial pressure, DC is needed to mitigate the deleterious effects of cerebral herniation (Figures 1A–C).

We recommend that DC is offered as early as possible when the patients develop clinical signs (such as a third nerve palsy) and radiological features of herniation due to large or expanding hemorrhagic or edematous infarcts (Figure 1D). Radiological features that prompt consideration of DC are large uncal herniation, midline shift ≥5 mm, and herniation induced hypodensity of the posterior cerebral artery territory. When it is anticipated that aggressive medical management is likely to fail and if there is insufficient time for anticoagulation to act by facilitating recanalization, DC should be done. Such a policy helps reduce the chances of herniation induced irreversible brain stem damage and posterior cerebral artery infarcts which can occur without much warning. In less severe cases, where there is no gross evidence of herniation, a trial of standard intensive care management of raised ICP with ventricular CSF drainage, osmotic agents, and transient hyperventilation may be done. Where there is uncertainty, the decision can be guided by ICP monitoring, but the insertion of a parenchymal or ventricular device must be done with normal coagulation parameters. Persistent ICP levels above 20 cm of CSF despite conservative management should also prompt consideration for DC (Figure 1D).

### Surgical Technique

A sufficiently large, unilateral hemicraniectomy, ideally centered on the site of the largest hematoma and venous infarct, allows expansive duraplasty with homologous or artificial material to reduce ICP. We believe that the recommendations for a large hemicraniectomy of 15 cm or greater for middle cerebral artery infarcts should also hold true for CVST since the reduction in ICP is the primary effect of DC. Infarcts affecting both anterior frontal lobes may be better dealt with using a bifrontal craniectomy, although there are no trials comparing both techniques. Evacuation of infarcted tissue is generally not recommended. However, spontaneous rupture of infarcts that typically occur at the site of dural opening warrants its removal. Medical management of cerebral edema should be continued in the postoperative period and may also be guided by ICP monitoring. There are as yet no definitive guidelines for ICP monitoring either before or after DC. The bone flap should be replaced once the brain swelling has subsided and this usually takes 3–6 months. We believe that cranioplasty should be done as early as reasonably possible to reduce the risk of complications of DC like subdural effusions, sunken skin flap syndrome, and hydrocephalus.

Since CVST is a rare cause of stroke and because of the ethical difficulties in delaying or not offering decompression when there is an obvious mass effect, large double-blinded randomized controlled studies seems difficult to conduct (25). Though the quality of evidence is low (Class IIb; Level of Evidence C), the intuitive need to perform DC in CVST is strong in select circumstances (6, 21). Meta-analysis and well-conducted systematic reviews combining data from multiple centers are useful when randomized controlled trials are unavailable. Unfortunately, even such studies evaluating the role of DC in CVST are sparse. Almost 10 years back, Coutinho et al. (26) and Lanterna et al. (27) independently published two reviews based on three previously published cases each where DC for large venous infarcts led to good outcomes. In the ISCVT study (28), the largest evaluation of its kind in CVST, 624 adult patients were registered. Most of the investigators of the trial were neurologists and only nine patients (1.4%) had a surgical intervention (29). Due to the low numbers of patients who underwent surgery, the role of DC was not analyzed. Seven years later, in 2011, the same investigators reviewed the role of DC with a combined retrospective registry and systematic review of 69 patients in 22 centers who had a surgical evacuation (30). During the last follow-up (median: 12 months), 15.9% of patients died and 5.8% of patients were severely dependent. The corresponding figures in their first report, wherein only 1.4% had undergone DC (*n* = 624) were 8.3 and 2.2%. Given the fact that only patients with malignant CVST underwent surgery (nine in ISCVT, 69 in the 2011 systematic review), the differences were comparable.

Over the years, multiple small observational studies suggest that surgery improves survival and produces acceptable outcomes even in patients with severe clinical conditions (8, 25, 30–32). The average death rate among patients treated with DC was 18.5%. The complete recovery rate was 30.7% and severe dependency rates were only 3.4% (21). The benefits are thought to be not only due to the prevention of progression of herniation but is also attributed to an improvement in the cortical venous collateral drainage that happens with the reduction in raised intracranial pressure. Unlike arterial infarcts, the variable patterns of apparent diffusion coefficient maps in MRI suggest that even large venous infarcts have a far better potential for recovery (24). Even in comatose patients and those with bilateral fixed pupils, DC seems beneficial, and leads to a good recovery in about one-third (8, 24) (Figure 1D).

Zuurbier et al. in a prospective cohort study of 10 DC patients reported a good clinical outcome in six patients, while two died (31). Aaron et al. (33), in a single center retrospective study on 44 patients undergoing DC reported a good outcome in 27 patients (61.4%) while nine patients died (20%). Theaudin et al. (24) retrospectively studied 12 patients with malignant cerebral edema out of 255 patients with CVST. All the four non-operated patients died, and all but one of the seven patient who underwent surgery survived and improved neurologically. The six survivors had a modified Rankin score (mRS) of 0 or 1 at 1 year. Authors also recommend that resection of infarcted tissue was not justified given the potential for recovery of venous infarction and suggests selective removal of large hematomas alone (24). Mohindra et al. in a retrospective study of 13 patients who underwent DC, reported a good outcome in all the 11 patients who survived. The two patients in their series who did not survive had a preoperative GCS <5 (34). In another retrospective study by Zhang et al. (32) of 58 patients who underwent DC, 46.6% had hemorrhage-dominated lesions and 56.9% had edema-dominated lesions. At 6 months, 56.9% of the patients attained a favorable outcome, while 13.8% died. Hemorrhage-dominated lesions and deep venous involvement cases had poorer outcomes.

We reviewed studies which were published after the last systematic review in 2011 by Ferro et al (30). Medline, PubMed, Google Scholar were used to identify studies reported in the English language with combinations of the following search terms: “cerebral sinus thrombosis,” “venous thrombosis,” and “craniectomy.” Only those studies which evaluated more than 15 patients who underwent DC and had follow-up assessment, were selected. Table 1 shows studies (2012 till the present) which evaluated the role of DC in CVST. Most of the single-center, high-volume studies were published from low-to-middle income countries, and includes two from the author's institution [see Table 1; (35, 37)] and it reflects the prevalence of uncorrected nutritional anemia and deficient perinatal care in general. Though endovascular services are available at the author's institution, DC was often required because the majority of the patients presented with impending herniation where the role of thrombolysis was limited. A total of 169 patients underwent DC for CVST in five studies and the mortality rate was 16.1%. A favorable outcome, defined as complete recovery or slight disability (mRS of 0–2, GOS of 5), could be calculated from three studies (*n* = 136) and such an outcome was achieved in 54.4% of the patients at the end of the follow-up period. Pre-operative ICP monitoring to guide the management plan was used only in one study but its effectiveness in decision making was not analyzed. Four studies favored DC in CVST with large hemorrhagic lesions causing midline shift and radiological features of intracerebral herniation. The results of our review are consistent with that by Ferro et al where mortality of 15.9% and a favorable outcome of 56.5% were reported. In addition to the obvious limitation that none of the studies had survival data from a control group who were managed conservatively, other shortcomings include the variations in the protocols employed, the myriad ways those protocols could have been escalated while the patients were being managed conservatively in different centers and the bias that is inherent to all retrospective uncontrolled studies. As mentioned before, the major strength of the present mini-review is that it included all the major studies till date which were published after the last systematic review in 2011.

**Table 1:** Major studies evaluating the role of decompressive craniectomy in cerebral venous sinus thrombosis published after the systematic review in 2011^a^.

**References**	**Type of study**	**Subjects** **(N^b^)**	**Study period**	**Mean GCS**	**Basis for DC^c^**	**Mean follow up** **(months)**	**Outcome**	**Comments**
Ferro et al. (30)	Systematic review, multicentric registry and review	69^d^	1998–2010	NA	Large hemispheric lesions and poor GCS	14.5	11 died. 39 patients recovered to mRS^7^ score 0 1 or 2.	Recommend DC
Vivakaran et al. (35)	Retrospective single center study	34	2006–2008	8.3	Clinical deterioration, Herniation syndrome	11.7	Four died. 14 recovered patients with GOS five	Recommend DC
Aaron et al. (33)	Retrospective single center study	44	2002–2011	NA	Volume of lesion and midline shift	25.5	Nine died. Three lost to follow up. Twenty seven patients had mRS core 0, 1 or 2	Recommend DC
Soyer et al. (36)	Retrospective single center study	16^e^	2002–2005	NA	Clinical deterioration. ICP monitoring was used in 8 patients	28	Five died during hospital stay. A detailed outcome assessment in treatment groups was lacking	For a similar CVST severity, craniectomy did not improve the outcome
Zhang et al. (32)	Retrospective single center study	58	2005–2015	6.7	Clinical deterioration, Herniation syndrome	6	Eight died. Thirty three patients attained a favorable outcome (mRS score of 0 in three patients, score of 1 in 13, and score of 2 in 17)	Recommend DC
Venkateswaran et al. (37)	Prospective cohort study	17	2015–2016	9 (median)	Clinical deterioration midline shift	18.6	One died. Two lost follow ups. Median mRS score of 1.5 in 14 patients	Improvement in regional cerebral oxygen saturation with DC

a *Only studies with a number of decompressive craniectomies more than 15 were selected*.

b *N stands for the total number of patients who underwent DC*.

c *Only the predominant reasons for DC are given in the table*.

d *Number of patients in the registry were 38, and 31 in Review: 45 patients underwent DC and the rest underwent other procedures*.

e *Total cases in the study were 47. DC, decompressive craniectomy; mRS, modifed Rankin score; CVST, cerebral venous sinus thrombosis*.

## Anticoagulation in CVST

In 1941, Lyons reported the first successful use of unfractionated heparin in two cases of cavernous sinus thrombosis (9). Presently, anticoagulation with hydration is the first-line treatment for CVST. Anticoagulation prevents propagation of the thrombus, hastens its spontaneous resolution, and aids in the prevention of deep vein thrombosis and embolism, without adversely promoting intracranial hemorrhage (ICH) (2, 19, 25, 38). ICH is not considered a contraindication for anticoagulation (9). Unfractionated heparin has to be given intravenously and it requires a dose adjustment based on activated partial thromboplastin time. Low-molecular-weight heparin (LMWH) is advantageous in that it can be administered as a subcutaneous injection based on body weight, and it has a more predictable pharmacokinetic profile (21) (Table 2). However, its effects are only partially reversed with protamine sulfate. The quality of evidence is too low to choose between the agents (21). LMWH is associated with lesser risk of new hematomas and seems to have better outcomes in ISCVT trial and other studies (25, 39, 40). It can be given in patients with normal renal function and those who do not require neurosurgical intervention (16, 39).

**Table 2:** Summary of various treatment modalities for CVST.

**Treatment modality**	**Treatment methods**	**Comments**
Anticoagulation	Intravenous • Unfractionated heparin • Low-molecular-weight heparin	The first line of treatment for CVST.
Fibrinolysis	Intravenous /Endovascular • Streptokinase • Urokinase	Small case series and prospective studies without a control group are available. Efficacy and safety are not established.
Thrombectomy	Endovascular • Mechanical thrombectomy • Suction thrombectomy • May be combined with fibrinolysis	Only a limited number of studies published. Further controlled trials are required to establish benefit.
Surgical intervention(s)	Open surgical thrombectomy	Few published case reports. With increasing access to endovascular modalities, microsurgical removal of thrombus is probably not indicated.
	Decompressive craniectomy	Class IIb; Level C Evidence. Indications include: 1. Clinical and radiological signs of herniation. 2. Persistently raised ICP refractory to medical management.

*CVST, cerebral venous sinus thrombosis; ICP, intracranial pressure*.

The time to restart therapeutic anticoagulation after DC is not clear (41, 42). Previous studies suggest that anticoagulation can be restarted after 24 to 48 h and some authors prefer to start with half the dosage for a period of 72 h (25, 31–33). Permanent anticoagulation is needed in those with prothrombotic states or with recurrent venous thrombosis (43). Other patients can be treated with oral Vitamin K antagonists for a period of 3–12 months (21, 44). There is limited safety data for oral anticoagulants such as Apixaban (45).

Systemic administration of fibrinolytic agents such as urokinase to recanalize thrombosed pathways has been attempted but strong evidence regarding its safety and efficacy is lacking (9). Endovascular thrombolysis may also be considered in patients who are unresponsive and deteriorating, despite aggressive medical treatment (10). Siddiqui et al., in their systematic review assessing mechanical thrombectomy with or without intrasinus thrombolysis suggested that this approach is safe (46). The overall death or dependency rate was 16% in patients who underwent mechanical thrombectomy, even though 47% of patients in this series were comatose or stuporous. The rate is comparable to that in the ISCVT study and indicates the safety of the approach (46). In cases where DC has no reasonable immediate role due to the absence of a life-threatening mass effect, patients may benefit from endovascular interventions (28, 47). Thrombolysis or anticoagulation for Cerebral Venous Thrombosis (TO-ACT trial) (48) study which sought to evaluate the role of endovascular thrombolysis was prematurely terminated due to futility.

## Decompressive Craniectomy in CVST in Different age Groups

### Pregnancy

Only case reports are available which describe DC in pregnant women. Patients might require a cesarean section for the safe delivery and resuscitation of the neonate (49). Puerperal CVST is more common and can be severe enough to warrant DC as a life-saving measure. Most of the studies indicate that future pregnancies are not contraindicated in women with a previous history of CVST (50). The absolute risk of recurrent venous sinus thrombosis associated with pregnancy in women who had a previous episode of CVST seems to be low, although the relative risk is much higher than the rate in the general population (50). Regardless of antithrombotic prophylaxis, the pooled estimate for recurrent CVST and non-cerebral venous thromboembolism associated with pregnancy was 9 per 1,000 pregnancies and 27 per 1,000 pregnancies, respectively (51). The avoidance of oral contraceptives and the use of anticoagulation prophylaxis during pregnancy dramatically reduced the probability of thrombosis recurrence in women (52).

### Pediatric CVST

In a study based on a Canadian pediatric stroke registry, neonates comprised of 43% of the children diagnosed with CVST, and 54% were younger than 1 year old (23). The increased risk in the neonates is attributed to multiple reasons such as the damage sustained by dural venous sinuses secondary to the molding of the skull bones during delivery, general prothrombotic state and dehydration (25). Infection is a major cause for CVST in children, and hypoxia is also thought to play a significant role in neonates (6, 23). Treatment with anticoagulants is generally considered to be safe, although studies are few (25, 53). In children over 2 years of age, a duration 3 to 6 months of anticoagulation should be tailored according to the cause (54). The indications and risk-benefit analysis of DC in pediatric CVST are not clear. DC is generally thought to be risky in neonates and young infants but may be cautiously considered in older children (55).

### CVST in the Elderly

CVST tends to be equally prevalent in older men and women. A headache as a presenting symptom is less common in the elderly (56). CVST should thus be added to the long list of disorders that cause depressed consciousness or mental changes in patients, and an extensive search must be done for such causes. In ISCVT, 8.2% of the patients were aged 65 years or older (22). The prognosis was worse with 49% of patients being dead or dependent at the end of the follow-up period. Due to an increased risk of thrombotic events, anticoagulation for more than 6 months may be warranted.

## Outcome

CVST has a favorable outcome when compared with other types of stroke. Due to increasingly early diagnosis and the widespread use of anticoagulation, the outcomes have been better than what existed half a decade ago (25, 57). The overall death rate is below 5% and about 80% of the patients make a complete recovery (mRS scores: 0–1) (21, 25, 38). However, mortality in severe cases with parenchymal lesions still remains as high as 35–50% (16, 50, 58). In ISCVT, 3.4% of patients died within the first month of thrombosis, 6.8% after 6 months and 8.3%, at the last follow up (median follow-up 16 months). Moderate to severe disability was reported in 5.1% of the patients (32). The role of recanalization of thrombosed veins in relation to the outcome is not very well established (39, 59, 60). In ISCVT, the main predictors of mortality within 30 days were male gender, age more than 37 years, seizure, mental status disturbance, GCS score <9, deep CVST, central nervous system infection, posterior fossa lesions and malignancy (8, 24). Patients older than 50 years, midline shift of more than 10 mm, total effacement of basilar cisterns, deep venous involvement, and bilateral lesions imply a poorer outcome in patients who underwent DC (8, 32).

## Conclusions

We recommend DC in select patients with medically intractable mass effect and raised intracranial pressure where herniation is an immediate risk. In less severe cases, therapeutic anticoagulation with LMWH and medical management of raised ICP seems reasonable. A large decompressive flap like the one recommended for middle cerebral artery infarcts for predominantly unilateral lesions or a bifrontal craniectomy for bifrontal infarcts are the surgical options.

## Author Contributions

RA wrote, edited and reviewed the manuscript. MG wrote and edited the manuscript. BD edited, critically reviewed the manuscript. DB and DS reivewed the manuscript. NS edited, reviewed the manuscript and prepared figures. All authors approved the final version.

### Conflict of Interest Statement

The authors declare that the research was conducted in the absence of any commercial or financial relationships that could be construed as a potential conflict of interest.
